# Comparative Evaluation of *Forsythiae Fructus* From Different Harvest Seasons and Regions by HPLC/NIR Analysis and Anti-inflammatory and Antioxidant Assays

**DOI:** 10.3389/fphar.2021.737576

**Published:** 2021-11-24

**Authors:** Qian Qu, Yuefei Li, Qi Dong, Shupeng Li, Hongliang Du, Zhihua Wang, Xiaopei Gong, Wenchang Zhang, Weijie Lv, Limin Chao, Mengjie Liu, Xinggang Tang, Shining Guo

**Affiliations:** ^1^ College of Veterinary Medicine, South China Agricultural University, Guangzhou, China; ^2^ Institute of Animal Health, Guangdong Academy of Agricultural Sciences, Guangzhou, China; ^3^ Guangdong Research Center for Veterinary Traditional Chinese Medicine and Natural Medicine Engineering Technology, Guangzhou, China; ^4^ Guangdong Laboratory for Lingnan Modern Agriculture, Guangzhou, China

**Keywords:** forsythiae fructus, harvest seasons, regions, anti-inflammatory, antioxidant

## Abstract

*Forsythiae Fructus* (FF), the dry fruit of *Forsythia suspensa* (Thunb.) Vahl, has a long history of use in traditional Chinese Medicine for its heat-clearing and detoxifying properties. It possesses clinical therapeutic effects and biological functions showing efficacy in handling different diseases. To investigate the FF differences in Henan, Shanxi, and Shaanxi in August and October, the surface morphology, mid-infrared and near-infrared spectrums, and HPLC were analyzed. Concurrently, the anti-inflammatory and antioxidant effects on LPS-induced J774A.1 cells were evaluated by western blot and RT-qPCR. The results showed that FF from different Harvest Seasons and Regions are provided with different microstructures and mid-infrared and near-infrared spectrums, and the levels of forsythiaside A and phillyrin of FF from Shanxi in August and phillygenin of FF from Shaanxi in August were the highest. Meanwhile, FF from Shanxi and Shaanxi in August markedly reduced the levels of inflammatory cytokines and mediators (TNF-α, IL-1β, NF-κB, and iNOS) and the protein expression levels of phosphorylated total IKKα/β and nuclear NF-κB. In August, SXFF and SAXFF also promoted the mRNA expression levels of HO-1 and NQO1 and the protein expression levels of HO-1 and nuclear Nrf2 and suppressed the protein expression levels of KEAP1. Spearman correlation analysis showed that phillygenin had a strong correlation with the protein expression on LPS-induced J774A.1 cells. In summary, our results showed that FF from harvest seasons and regions contributed to the distinct differences in microstructure, the mid-infrared and near-infrared spectrums, and compound content. More importantly, FF from Shanxi and Shaanxi in August showed marked anti-inflammatory and antioxidant activities, but with some differences, which may be because of different contents of phillygenin and phillyrin of lignans in FF.

## Introduction

*Forsythiae Fructus* (FF) is the dry fruit of *Forsythia suspensa* (Thunb.) Vahl in the family Oleaceae, which has been widely used as an antipyretic and antidotal herb in Traditional Chinese Medicine (TCM) (named Lianqiao in Chinese) for thousands of years. The TCM characteristics of FF are summarized as a bitter flavor with a mildly cold nature and lung, heart, or intestinal meridian distribution; these characteristics are parallel to the characterization of anti-inflammatory TCM (Lee et al., 2018). FF is used to treat pyrexia, gonorrhea, carbuncles, and erysipelas in Shennong’s Herbal, and it is also included in many TCM prescriptions, which are used to treat influenza, hyperlipidemia, cardiovascular, pneumonia, and so on. Indeed, more than 40 TCM prescriptions containing FF are listed in the Chinese pharmacopoeia (Bao et al., 2017; Tsai et al., 2018; Zhang et al., 2021).

According to the harvest period, two kinds of FF are selected: one is a green, indehiscent, and nearly ripe fruit that is harvested in August and September and named Qingqiao in Chinese; the other is a yellow, dehiscent, and fully ripe fruit that is harvested in October and named Laoqiao (Wang et al., 2018). Both of them serve as official sources of FF; however, Qingqiao is used more frequently in TCM prescriptions (Jia et al., 2015; Fang et al., 2018). FF is predominantly produced in the Hebei, Shaanxi, Shanxi, Shandong, Anhui, Henan, Hubei, Jiangsu, and Sichuan Provinces (Wang et al., 2018). In TCM, genuine Chinese medicine has better efficacy and disease treatment effects, which may be due to their different origins, resulting in different chemical substances even though they are the same kind of herbs (Zhu et al., 2019; Luo et al., 2021). FF contains many chemical components, such as forsythoside A and B (phenylethanolamine), phillyrin, and phillygenin (lignans), which have been reported to exhibit multiple biological activities (Zhang et al., 2020b; Hu et al., 2020; Jiang et al., 2020; Wang et al., 2021b). What are the distinctions between FF from different producing areas and harvest time? What kinds of changes happen to the function of FF caused by different producing areas and harvest time? Previous studies have shown that the obvious distinctions in compounds between green and ripe FF may be the main reason for their different anticancer activity (Bao et al., 2017). Additionally, the Jia showed that green and ripe FF have distinct chemical compositions based on NMR metabolic profiling (Jia et al., 2015).

Near-infrared (NIR) spectroscopy is a promising method that has been widely used as a rapid and non-destructive technique for qualitative and quantitative analysis of traditional Chinese medicine (TCM) (Wang et al., 2017; Wang et al., 2019). A variety of methods have been applied for the identification of TCM, such as mid-infrared (MIR) spectroscopy, high-performance liquid chromatography, mass spectrometry, chemometrics, and so on (Chen et al., 2016; Qu et al., 2017; Zhao et al., 2020). For instance, Sun et al. identified the genuine and adulterated Pinellia ternate by MIR and NIR spectroscopy, and Chen et al. evaluated the decoction pieces of *Rhizoma Atractylodis Macrocephalae* by near-infrared spectroscopy coupled with chemometrics (Chen et al., 2019; Sun et al., 2019).

Previous studies have shown that FF is widely used to treat lipopolysaccharide (LPS)-induced inflammation and oxidation (Lee et al., 2018; Shao et al., 2021). In response to LPS, macrophages produce NO, which is synthesized by iNOS, increasing the level of nuclear NF-κB as well as pro-inflammatory cytokines such as TNF-α, IL-1β (Heo et al., 2018; Hung et al., 2019). It also activates the Nrf2 signaling pathway, resulting in the secretion of antioxidant enzymes such as HO-1 and NQO1 (Lim et al., 2020).

In the present study, the differences in the microstructure and mid-infrared and near-infrared spectrum were observed in three chemical compositions of *Forsythia Fructus* collected from Henan, Shanxi, and Shaanxi in August and October. We, therefore, aimed to further explore whether these differences lead to changes in their anti-inflammatory and antioxidant effects and to provide a certain chemical basis for the clinical use of FF in August and October from Henan, Shanxi, and Shaanxi.

## Materials and Methods

### Reagents and Antibodies

Forsythoside A (purity>98%), phillyrin (purity>98%), and phillygenin (purity>97%) were purchased from Dalian Meilun Biotechnology (China), and methanol (purity>99%) and acetonitrile (purity>99%) were obtained from Thermo Fisher Scientific—CN. Antibodies specific to *α*-tubulin, *β*-Actin, TBP, HO-1, Nrf2, KEAP1, P-NF-κB p65, *p*-IKKα/*β* were purchased from Cell Signaling Technology and Lipolysaccharide (LPS) and Penicillin-Streptomycin from Sigma-Aldrich.

### Preparation of the FF Methanol Extract

In total, 180 FF samples (30 g) were acquired from apiculture producers from the Shanxi, Shaanxi, and Henan Provinces in China in August and October. In total, 180 samples were from different times and different provinces [FF in August from Shanxi Province (SXFF-A), October from Shanxi Province (SXFF-O), August from Shaanxi Province (SAXFF-A), October from Shaanxi Province (SAXFF-O), August from Henan Province (HNFF-A), and October from Henan Province (HNFF-O)]. FF samples were broken to pieces and sieved through 60 pieces of mesh, dissolved in methanol, and extracted by ultrasound for 30 min. On the next day, samples were extracted again by ultrasound for 30 min and then centrifuged at 3,000 rpm. The upper methanol layer was filtered using a 0.22 μm PVDF membrane and stored at −80°C.

### Surface Morphology of FF

The FF were powdered and screened through 60 meshs, and evenly poured on to tape. After gold-plating, a scanning electron microscope (SEM) (SUPRA 55 VP, Zeiss) was used to collect samples at an accelerating voltage of 5.00 kV with a magnification of 4,500 times.

### Mid-infrared of FF

After 60 mesh screening, the 1 mg FF samples were mixed with 100 mg KBr powder, and the mixture was ground to less than 2 μm and put into an infrared tablet press with 20–24 MPa for approximately 1 min form potassium bromide tablets. The analysis was performed in the frequency range of 4,000 cm^−1^ to 400 cm^−1^ using a Vertex 70 FT-IR spectrometer (Brooke Biotechnology, Germany).

### HPLC Analysis of FF

Analysis of constituents in FF was performed on an 1525-2707-2489 series HPLC system (Waters, United States) equipped with a binary solvent manager, sample manager, column compartment, UV detector with 280 nm, and LC-Solution software. The separation was performed on an Agilent TC-C_18_ column (5 μm, 4.6 × 250 mm). The mobile phase consisted of water containing 0.1% acetic acid (A) and acetonitrile (B). The linear gradient was as follows: 0–30 min, 10–25% B; 30–45 min, 25–75% B; and 45–50 min, 75% B at a flow rate of 1.0 ml/min. The column temperature was maintained at 28°C and the injection volume was 10 μL (Lee et al., 2018). Chemicals used as a standard are Forsythoside A, phillyrin, and phillygenin. The constituents were identified by comparison of their retention times to those of standard compounds under identical analysis conditions and the UV spectra with our in-house DAD library.

### Acquisition of Near-Infrared Spectrum

The NIR spectrum of FF was measured by an Antaris II FT-NIR spectrometer (Thermo Fisher Scientific, Verona, United States), with a wavenumber range from 10,000 to 4,000 cm^−1^, scanning times of 64, and a resolution of 8 cm^−1^. The signals were generated in reflectance (%R) mode and showed using log 1/R. The NIR spectrum was recorded in triplicate for each sample, and the average spectrum was used in the data analysis. Analysis of NIR spectrum was performed with TQ Analyst 9.0 software.

### Cell Culture

J774A.1 cells purchased from the American Type Culture Collection were cultured according to a previously described method (Alizadeh et al., 2021). Cells were cultured in high glucose DMEM containing 10% fetal bovine serum, 100 U/ml penicillin, and 100 μg/ml streptomycin (Gibco; Thermo Fisher Scientific, Inc.) in a humidified incubator at 37°C with 5% CO_2_.

### Measurement of Cell Viability

Cell viability was measured by the MTT assay, which is described in the previous research (Hung et al., 2019). Briefly, the cells were seeded in a 96-well plate at a density of 2 × 10^4^ cells/well, treated with 1, 0.5, 0.2, 0.1 and 0.05 mg/ml SXFF-A and 1, 0.5, 0.2, 0.1 and 0.05 mg/ml SAXFF-A for 24 h. After incubation, the supernatant was removed, and then 100 μL of MTT solution (0.5 mg/ml) was added to each well and incubated for 4 h at 37°C. Next, the cell culture supernatant was removed and the resulting formazan crystals were dissolved in 100 μL DMSO. The absorbance was evaluated at 540 nm using a Multi-Mode Microplate Reader (Thermo Fisher Scientific, Inc.).

### Measurement of NO Production

The levels of NO in the J774A.1 cells and the supernatant were performed with Griess Reagent I and II (Beyotime Co., Ltd.). The cells were seeded in a 96-well plate at a density of 2 × 10^4^ cells/well, treated with or without 0.5, 0.2, and 0.1 mg/ml SXFF-A and 0.5, 0.2, and 0.1 mg/ml SAXFF-A for 0.5 h followed by the addition of LPS (1 μg/ml) for 24 h. After incubation, the cell culture supernatant was transferred to 96-well, and the remaining cells were cleaved with Cell lysis buffer for Western and IP (Beyotime Co., Ltd.) to measure NO levels in supernatant and cells according to the manufacturer’s instructions. Briefly, 50 μL cell culture supernatant and 50 μL centrifuged cell lysis supernatant was mixed with 50 μL Griess Reagent I and II respectively for 10 min, and the absorbance was evaluated at 540 nm using a Multi-Mode Microplate Reader.

### Isolation of Total RNA and RT-qPCR

J744A.1 cells were plated onto a 12-well plate at a density of 4 × 10^5^ cells/well, treated with or without 0.5, 0.2, and 0.1 mg/ml SXFF-A and 0.5, 0.2 and 0.1 mg/ml SAXFF-A for 0.5 h, and then had LPS (1 μg/ml) added to them for 12 h. Total RNA was extracted from collected cells using FastPure^®^ Cell/Tissue Total RNA Isolation Kit (Vazyme Biotech Co., Ltd.) according to the manufacturer’s instructions. cDNA was synthesized with total RNA (1 μg) using HiScript III RT SuperMix for qPCR (+gDNA wiper) (Vazyme) and subjected to real-time quantitative PCR (RT-qPCR) amplification using the ChamQ universal SYBR qPCR Master Mix (Vazyme) in a QuantStudio^®^5 (Thermo Fisher Scientific, Inc.). The primer sequences are shown in Table 1, and the data were analyzed and expressed as relative gene expression to *β*-Action using the 2^−△△^CT method.

**TABLE 1:** The gene sequence for RT-qPCR.

Target gene	Direction	Sequence (5–3′)
IL-1β	Forward	AGG​CAG​GCA​GTA​TCA​CTC​ATT​G
	Reverse	CGT​CAC​ACA​CCA​GCA​GGT​TAT​C
TNF-α	Forward	CTC​ACA​CTC​ACA​AAC​CAC​CAA​G
	Reverse	CAA​TGA​CTC​CAA​AGT​AGA​CCT​GC
iNOS	Forward	CTG​CCA​GGG​TCA​CAA​CTT​TAC
	Reverse	CAG​CTC​AGT​CCC​TTC​ACC​AA
NF-κB	Forward	GGA​GGC​ATG​TTC​GGT​AGT​GG
	Reverse	GCGATGGGTTCCGTCTTG
HO-1	Forward	GCT​GGT​GAT​GGC​TTC​CTT​GT
	Reverse	GCA​TAG​ACT​GGG​TTC​TGC​TTG​TT
NQO1	Forward	AGG​ACG​CCT​GAG​CCC​AGA​TA
	Reverse	CTG​GAA​AGG​ACC​GTT​GTC​GTA​C
β-Actin	Forward	TGC​TGT​CCC​TGT​ATG​CCT​CTG
	Reverse	CTG​TAG​CCA​CGC​TCG​GTC​A

### Western Blot Analysis

J744A.1 cells were plated onto a 6-well plate at a density of 6 × 10^5^ cells/well, treated with or without 0.5, 0.2, and 0.1 mg/ml SXFF-A and 0.5, 0.2, and 0.1 mg/ml SAXFF-A for 0.5 h, then had LPS (1 μg/ml) added to them for 12 h. The total protein and nuclear protein were extracted using Enhanced RIPA Lysis buffer with a 1 mM PMSF and phosphatase inhibitor cocktail (Beyotime) and a Nuclear Extract kit (EMD Millipore Corp), respectively, according to the manufacturer’s instructions. The concentrations of protein were determined by a BCA protein assay kit (Beyotime) according to the manufacturer’s instructions. Then, briefly, the protein (10 μg/lane) was separated with SDS-PAGE and transferred to a PVDF membrane, followed by blocking with 5% non-fat milk. After blocking, they were incubated with primary antibodies at 1:1,000 dilution in primary antibody dilution buffer overnight at 4°C and then incubated with the following HRP-conjugated secondary antibodies (1:10,000) in TBST at room temperature for 1 h, and immune-reacted bands were detected with enhanced chemiluminescence reagents. The relative density of the western blot bands was determined using the BLT GelView 6,000 Pro software.

### Statistical Analysis

SPSS software (version 26.0), Origin 2021, and GraphPad (version 8.0) were used for statistical analysis. Statistical comparisons were assessed by one-way analysis (ANOVA), followed by Least significant difference (LSD) test. All experiments were performed at least three times independently.

## Results

### Surface Morphology of FF by SEM

**FIGURE 1:**
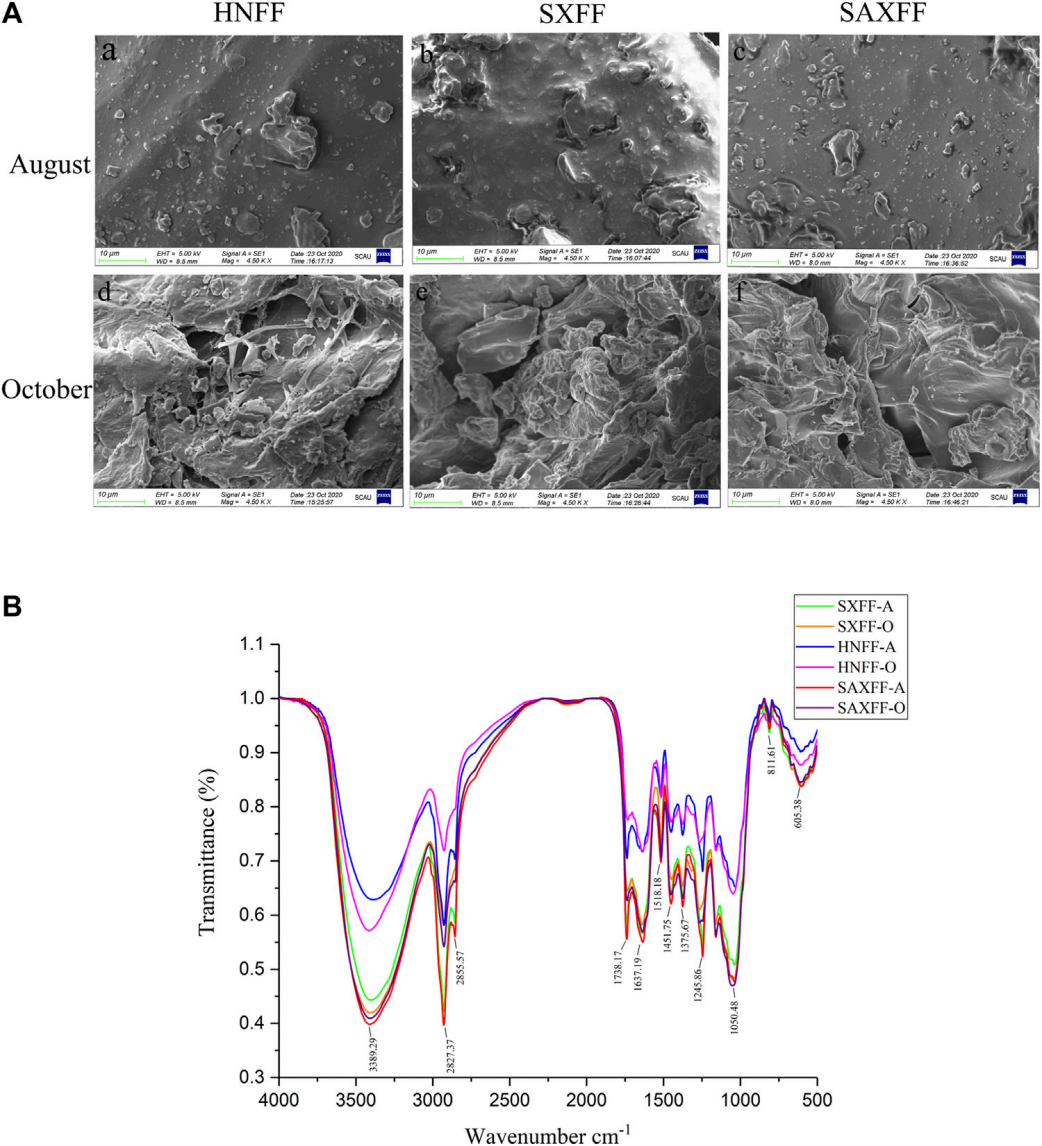
Scanning electron microscope and Mid-infrared spectra of FF. **(A)** Scanning electron microscope of FF from Henan Province (HNFF), Shanxi Province (SXFF), and Shaanxi Province (SAXFF) in August and October. **(B)** Mid-infrared (MIR) spectra of FF from Shanxi Province in August (SXFF-A), Shanxi Province in October (SXFF-O), Henan Province in August (HNFF-A), Henan Province in October (HNFF-O), Shaanxi Province in August (SAXFF-A), and Shaanxi Province in October (SAXFF-O).

The obtained SEM micrographs of FF from different harvest seasons and regions showed obvious differences in the surface morphologies, as shown in Figure 1A. The SEM images indicated that the surface structure of FF from the same regions in October was looser and had a more net-like structure than that in August, whereas no significant differences were observed among the three regions in August and October. There were also more pore-like structures on the surface of SXFF-A compared with that in SAXFF-A and HNFF-A (Figure 1A a–c).

### MIR Spectra of FF

The MIR spectra revealed that FF from different harvest seasons and regions had the same functional groups through the absorption bands of the phytocompounds (Figure 1B). Bands for FF were obtained at peak 3,389 cm^−1^, 2,927.37 cm^−1^,1738.17 cm^−1^, 1,637.19 cm^−1^, 1,245.86 cm^−1^, 1,032.27 cm^−1^, and so on. The results showed that FF in different harvest times and regions have the same absorption peaks and different transmittance. This suggested that FF were provided with identical major functional groups, but different MIR spectra transmittance, indicating that FF from different regions and harvest times had similar compounds, yet may have varied in the content of the compound.

### NIR Spectra of FF

**FIGURE 2:**
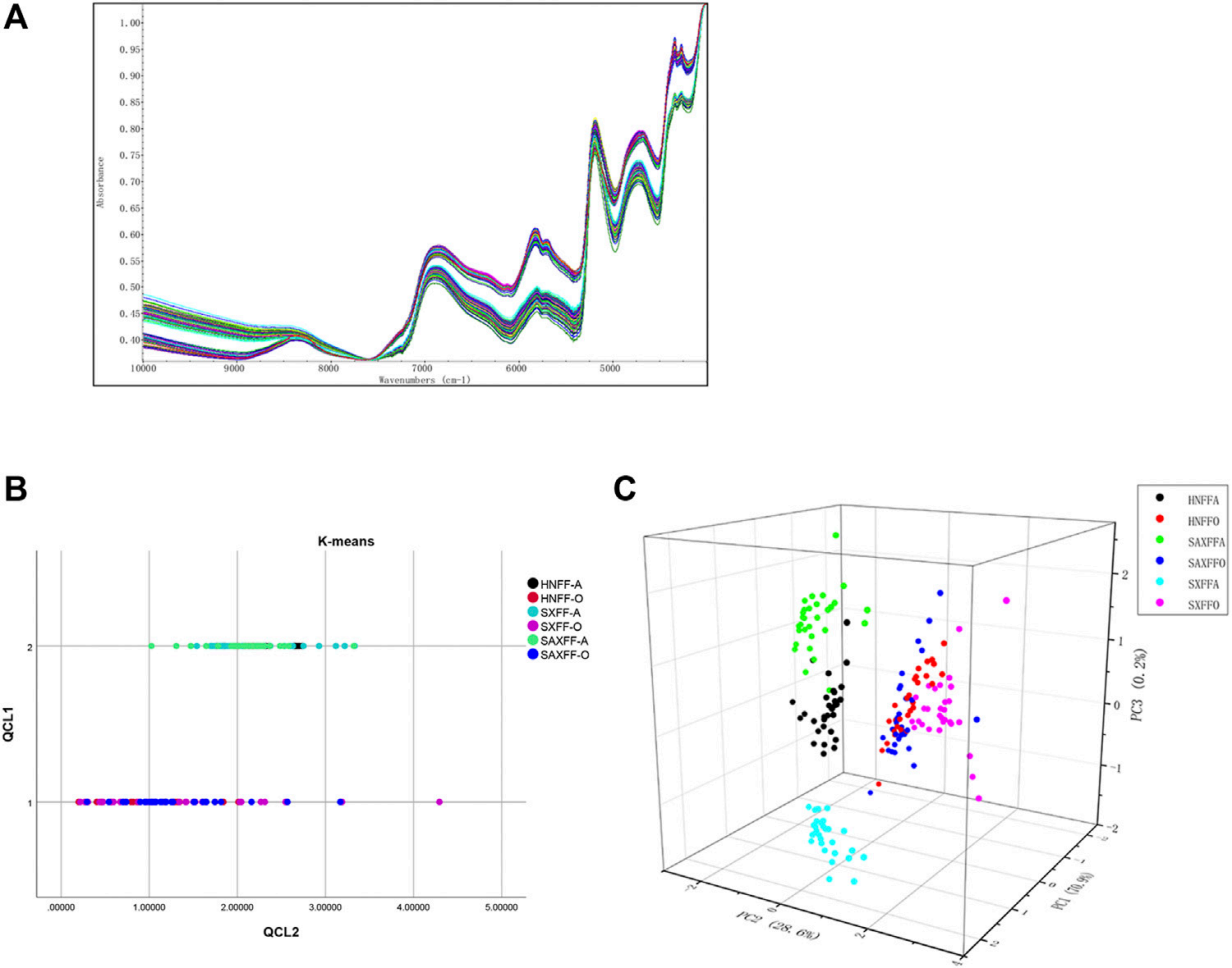
Analysis of NIR spectrum of FF from different harvest seasons and regions. **(A)** NIR spectrum images of 180 FF in the frequency range of 10,000 to 4,000 cm-1 with 8 cm-1 increments. **(B)** The K-means cluster analysis of NIR spectrum of 180 FF from different harvest seasons and regions by SPSS software (version 26.0). QCL1 is the number of clusters and QCL2 is the distance from the sample to the cluster center. **(C)** 3-dimensional graph of NIR spectrum of 180 FF based on PCA. PC1, PC2, and PC3 are the three main components after dimension reduction.

The NIR spectrum of 180 FF samples from different gathering times and localities is shown in Figure 2, which shows that there is a significant distinction among them. FF samples were recorded by NIR spectra from 10,000 to 4,000 cm^−1^ with a resolution of 8 cm^−1^ (Figure 2A). Cluster analysis is used to analyze the similarity of the NIR spectrum of 180 FF samples by clustering samples based on their intimacy. K-means clustering analysis is an iterative clustering analysis method. Firstly, all data are pre-divided into K groups, and K objects are randomly selected as the initial cluster center. Then, the distance between each object and each seed cluster center is calculated, and each object is assigned to the nearest cluster center. According to the results of K-means cluster analysis, 180 FF samples were divided into two clusters: FF from Hehan, Shanxi, and Shaanxi in August and in October. The different producing areas of FF were not distinguished, indicating that the effect of harvest time on FF was more obvious than that of regions (Figure 2B).

Principal component analysis (PCA), which reduces its dimensionality into several main components, is applied to analyze the NIR spectrum of the 180 FF samples. PCA is a dimensionality reduction method that converts a large quantity of data into a few comprehensive indicators, reducing the dimension of observation and obtaining the most important information. In this study, the NIR spectrum of PCA based on variance decomposition was projected into the new coordinate system, and the principal components (PC1, PC2, and PC3) take the eigenvalues that can reflect the maximum variance value. Figure 2C showed the PC1, PC2, and PC3 loading plots of different FF PCA models that corresponded to 70.9, 28.6, and 0.2% of the variance. The three-dimensional scores of PCA showed there was a significant difference in the distribution of the FF between August and October; Henan, Shanxi, and Shaanxi in August were also distinguished, whereas there was no significant difference in October of the FF from Henan, Shanxi, and Shaanxi (Figure 2C).

Taken together, the K-means clustering and PCA of NIR spectrum showed that FF from different producing areas and harvest seasons had obvious characteristics of the harvest season. Meanwhile, FF in August showed obvious characteristics of producing area, while FF from different producing areas in October is not distinguished. These results indicated that the impact of harvest season on FF was significantly greater than that of producing areas.

### The Forsythiaside A, Phillyrin, and Phillygenin of FF

**FIGURE 3:**
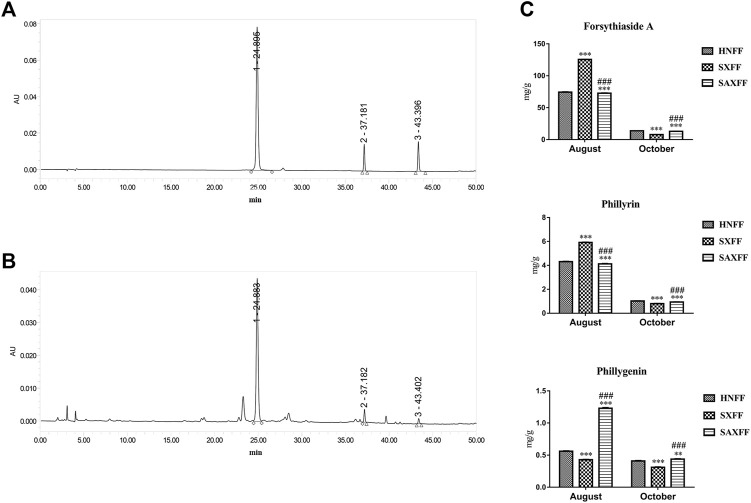
The contents of forsythiaside A, phillyrin, and phillygenin of FF by HPLC. **(A)** Standard chemicals of forsythiaside A, phillyrin, and phillygenin, **(B)** the methanol extract of Forsythia suspense by HPLC, and peak numbers indicate the following compounds, peak 1 is Forsythiaside A, peak 2 is phillyrin and peak 3 is phillygenin. **(C)** The levels of forsythiaside A, phillyrin, and phillygenin of FF from Henan, Shanxi, and Shaanxi in August and October.

To investigate the differences in the surface morphology, MIR spectra transmittance, and the NIR spectra of FF from different harvest seasons and regions, the contents of forsythiaside A, phillyrin, and phillygenin were determined by HPLC. The chromatograms of the forsythiaside A, phillyrin, and phillygenin from standards and samples were shown in Figures 3A,B. And the contents of the three components showed that different producing areas and harvest time played obvious effects on the FF (Figure 3C). Compared with HNFF, the forsythiaside A, phillyrin, and phillygenin of SXFF and SAXFF in August was markedly improved, while that of HNFF and SAXFF was significantly higher than that of SXFF in October. The level of forsythiaside A and phillyrin in SXFF-A was the highest and the phillygenin in SAXFF-A was the highest, which indicated that the SXFF-A and SAXFF-A perhaps had the highest constituents basis.

### Anti-inflammatory Activity of FF on LPS-Induced J774A.1 Cells

**FIGURE 4:**
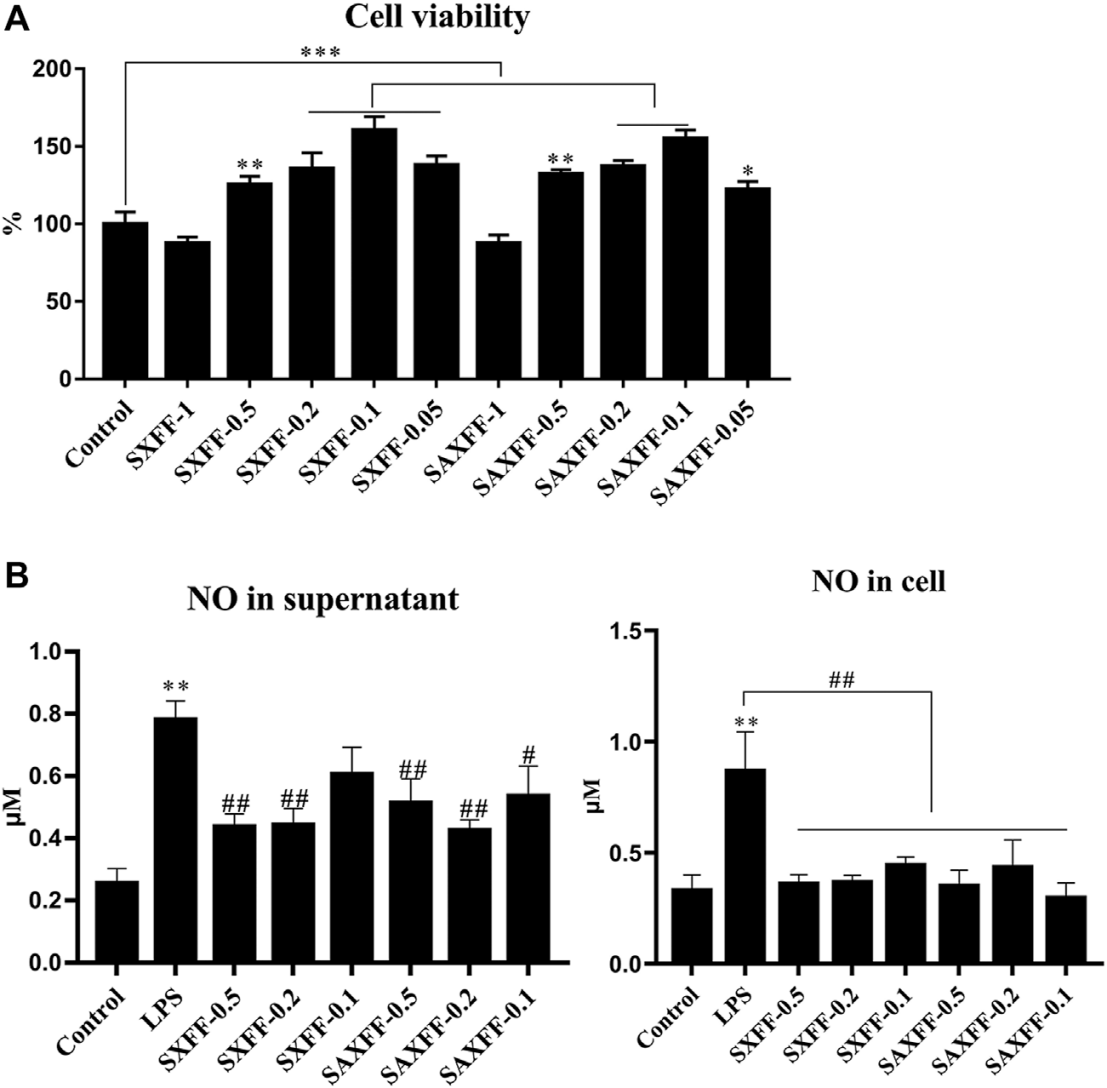
The cell viability and NO levels of FF on LPS-induced J744A.1 cells. **(A)** LPS-induced J744A.1 cells were treated with FF from Shanxi and Shaanxi in August for 24 h, cell viability was detected by MTT. The concentration of FF from Shanxi and Shaanxi were 1, 0.5, 0.2, 0.1, and 0.05 mg/ml, respectively. **(B)** The NO levels in supernatant and cells of LPS-induced J744A.1 cells treating with FF were measured by Griess reaction. SXFF-0.5, SXFF-0.2, and SXFF-0.1 is FF from Shanxi in August with concentration of 0.5, 0.2, and 0.1 mg/ml, and SAXFF-0.5, SAXFF-0.2, and SAXFF-0.1 is FF from Shaanxi with concentration of 0.5, 0.2, and 0.1 mg/ml, respectively. ^*^ *p* < 0.05, ^**^ *p* < 0.01, and ^***^ *p* < 0.001 compared with the Control group; ^#^ *p* < 0.05, ^##^ *p* < 0.01, and ^###^ *p* < 0.001 compared with the LPS group.

**FIGURE 5:**
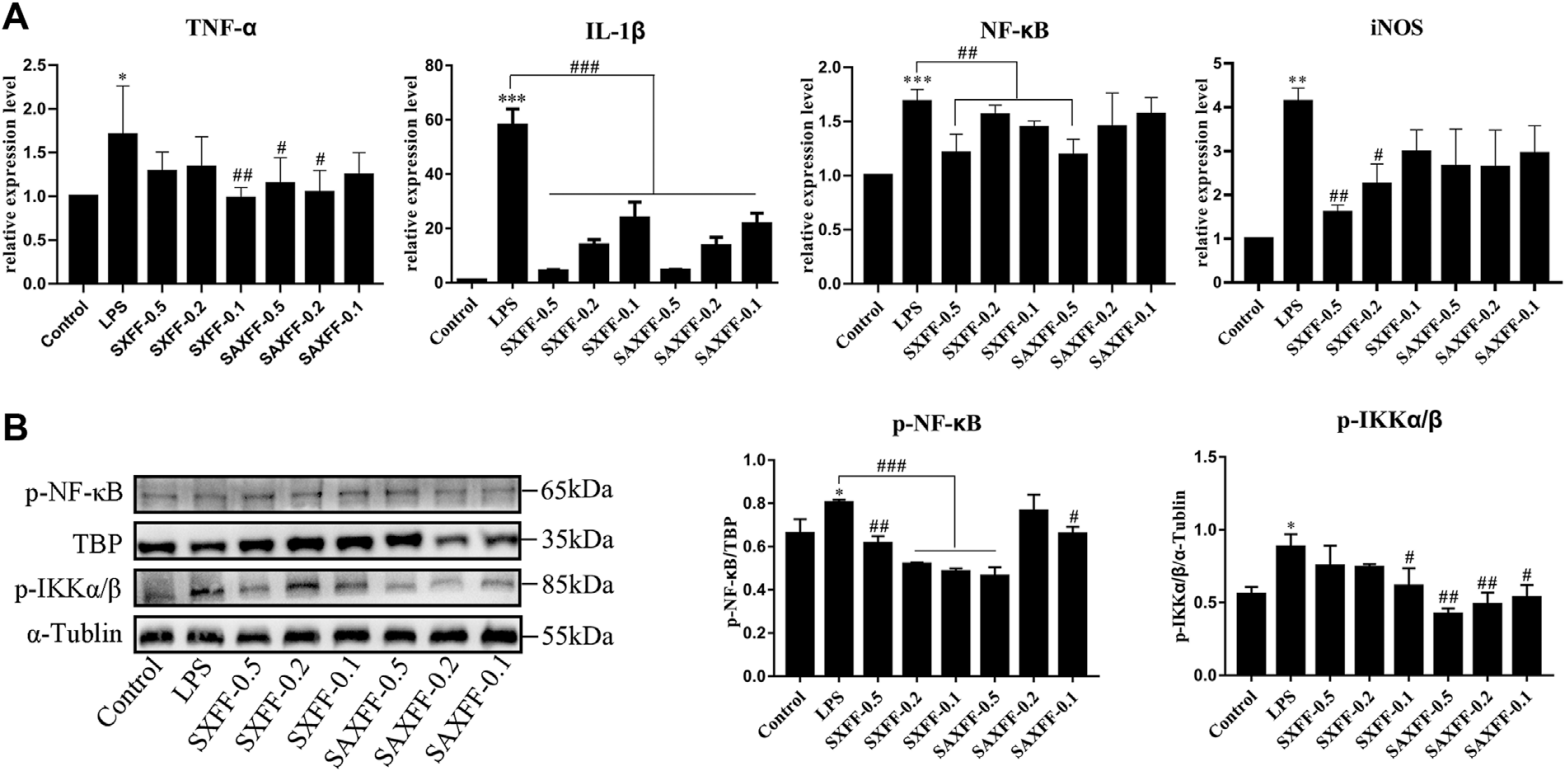
Anti-inflammatory activity of FF from Shanxi and Shaanxi in August on LPS-induced J774A.1 cells. **(A)** The mRNA expression levels of TNF-α, IL-1β, NF-κB, and iNOS were measured by RT-qPCR. **(B)** the protein expression levels of phosphorylated total IKKα/β and nuclear NF-κB were detected by Western Blot. ^*^ *p* < 0.05, ^**^ *p* < 0.01, and ^***^ *p* < 0.001 compared with the Control group; ^#^ *p* < 0.05, ^##^ *p* < 0.01, and ^###^ *p* < 0.001 compared with the LPS group.

Based on the high levels of forsythiaside A, phillyrin, and phillygenin by HPLC, SXFF, and SAXFF in August were selected to treat J774A.1 cells. The results of the MTT assay suggested that 0.5 mg/ml, 0.2 mg/ml, and 0.1 mg/ml of SXFF and SAXFF in August were not toxic to J774A.1 cells (Figure 4A), which indicated that the effects of SXFF and SAXFF on J774A.1 cells were not due to their cytotoxicity. The NO levels in supernatant and cells of LPS-induced J774A.1 cells were significantly reduced by SXFF and SAXFF (Figure 4B). In this study, the anti-inflammatory activity of FF on LPS-induced J774A.1 cells was investigated. As shown in Figure 5A, the relative expression levels of inflammatory cytokines and mediators, namely, TNF-α, IL-1β, NF-κB, and iNOS were markedly downregulated by SXFF and SAXFF (August) in LPS-induced J774A.1 cells. Moreover, SXFF obviously downregulated the relative expression levels of IL-1β and iNOS mRNA, and SAXFF significantly attenuated the expression of IL-1β and NF-κB in a dose-dependent manner.

NF-κB is a transcription factor in promoting inflammation, so we investigate whether the anti-inflammatory effect of SXFF and SAXFF is related to the suppression of NF-κB. Western blot analysis was performed to analyze the protein expression levels of phosphorylated total IKKα/β and nuclear NF-κB. As shown in Figure 5B, the levels of *p*-IKKα/β and nuclear p-NF-κB were markedly increased by LPS and remarkably decreased by SXFF and SAXFF in LPS-induced J774A.1 cells, and the levels of *p*-IKKα/β were decreased by SAXFF in a dose-dependent manner.

Taken together, these results showed that SXFF and SAXFF suppressed the relative expression levels of TNF-α, IL-1β, NF-κB, and iNOS mRNA and the protein expression levels of *p*-IKKα/β and nuclear p-NF-κB, suggesting that SXFF and SAXFF have evident anti-inflammatory function *in vitro*, and the anti-inflammatory effects may be related to suppressing the NF-κB signaling pathway.

### Antioxidant Activity of FF on LPS-Induced J774A.1 Cells

**FIGURE 6:**
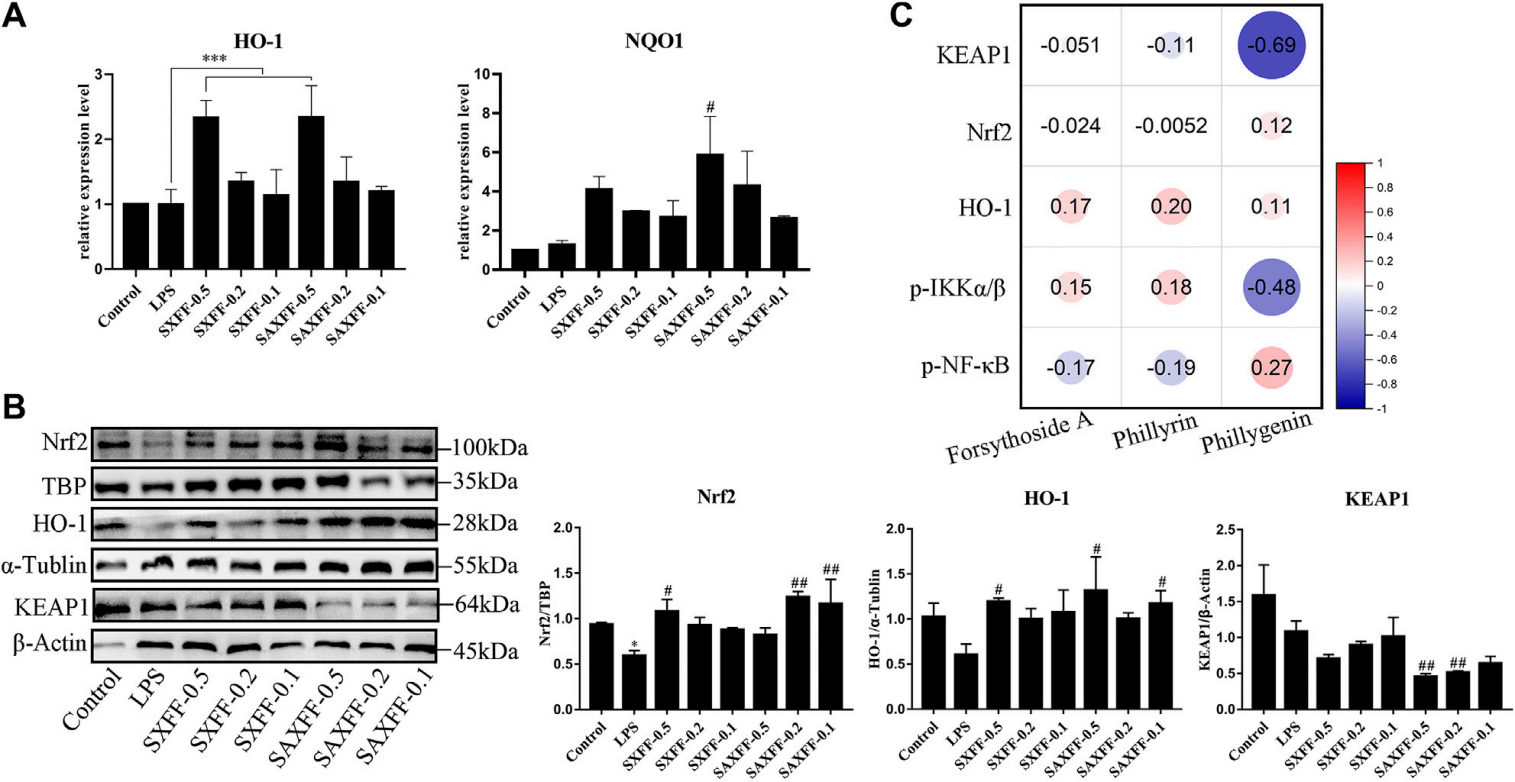
Antioxidant activity of FF from Shanxi and Shaanxi in August on LPS-induced J774A.1 cells. **(A)** The mRNA expression levels of HO-1 and NQO1 by LPS-induced J774A.1 cells treated with FF were measured by RT-qPCR. **(B)** The protein expression levels of total HO-1, KEAP1, and nuclear Nrf2 were assayed via Western Blot. ^*^ *p* < 0.05, ^**^ *p* < 0.01, and ^***^ *p* < 0.001 compared with the Control group; ^#^ *p* < 0.05, ^##^ *p* < 0.01, and ^###^ *p* < 0.001 compared with the LPS group. **(C)** Spearman correlation analysis between the levels of forsythoside A, phillyrin, and phillygenin and the protein expression levels of KEAP1, Nrf2, HO-1, *p*-IKKα/β, and p-NF-κB by Origin software. The size of the circle represents the correlation, red represents a positive correlation, and blue represents a negative correlation.

Nrf2 is an important transcription factor regulating the cellular oxidative stress response, and also a central regulator maintaining intracellular redox homeostasis. Therefore, we investigated how SXFF and SAXFF regulate Nrf2 signaling to exert an antioxidant role, and whether there is a difference in antioxidant effect between SXFF and SAXFF. As shown in Figure 6A, the mRNA expression levels of HO-1 were significantly increased by SXFF and SAXFF with a dose of 0.5 mg/ml, and the NQO1 were markedly increased by SAXFF with a dose of 0.5 mg/ml in LPS-induced J774A.1 cells.

To further determine the antioxidant effect of SXFF and SAXFF, Western blot analysis was performed to analyze the protein expression levels of total HO-1, KEAP1, and nuclear Nrf2. As shown in Figure 6B, SXFF and SAXFF significantly improved the protein expression levels of total HO-1 and nuclear Nrf2. Meanwhile, the protein expression levels of total KEAP1were obviously reduced by SXFF and SAXFF in a dose-dependent manner, and the levels of KEAP1 in SAXFF were lower than that in SXFF.

Collectively, these results showed that SXFF and SAXFF promoted the mRNA expression levels of HO-1 and NQO1 and the protein expression levels of HO-1 and nuclear Nrf2 and suppressed the protein expression levels of KEAP1, suggesting that the antioxidant activity of SXFF and SAXFF on LPS-induced J774A.1 cells is correlated with the Nrf2 signaling, and the antioxidant activity in SAXFF was stronger than that in SXFF.

### The Correlation Analysis

In view of the different contents of forsythoside A, phillyrin, and phillygenin in FF from different localities, we analyzed the correlation between them and the anti-inflammatory and antioxidant effects of FF based on Spearman correlation. As shown in Figure 6C, the results showed that the correlation between the levels of phillygenin and the protein expression levels of KEAP1, Nrf2, and *p*-IKKα/β was higher than that of forsythoside A and phillyrin, whereas the correlation between the phillyrin and the protein of HO-1 and p-NF-κB was higher than that of forsythoside A and phillygenin. Phillyrin and phillygenin belong to bicyclo lignans, which indicates that the anti-inflammatory and antioxidant effects of FF may be mainly exerted by the lignans.

## Discussion

*Forsythiae fructus* (FF), which includes forsythoside A, forsythoside B, phillyrin, phillygenin, etc., is widely used as Chinese herbal medicine for heat-clearing and detoxification purposes and appears in more than 100 kinds of TCM (Chen et al., 2016; Zhang et al., 2020b; Wang et al., 2021a). In fact, previous studies have shown that FF has anti-inflammatory, antiviral, anticancer, and other therapeutic effects (Ko et al., 2005; Long et al., 2020; Wang et al., 2021b). In TCM, there are many distinctions between geo-authentic and non-authentic producing areas, and the picking time also has marked impacts on herbs. FF has many producing areas, including Henan, Hebei, Shanxi, Shaanxi, and so on. In August or September, FF is harvested when it is green and immature, which is called Qingqiao, and in October, the yellow and mature FF is called LaoQiao. In the present study, FF collected in August and October from Henan, Shanxi, and Shaanxi was examined to identify the distinctions in the surface morphology, compound content, NIR and MIR spectrum, and the anti-inflammatory and anti-oxidation effects of FF.

Scanning electron microscope (SEM) is a momentous technique for the determination of morphological parameters and is performed to analyze the surface morphology of FF from different harvest seasons and regions, providing visual evidence (Yao et al., 2019; Mitsuwan et al., 2020). In the present study, the SEM images showed that FF in October has more loose and reticular structure than that in August. And the data of MIR spectra demonstrated that FF from different picking times and areas had similar compounds, but different contents of some components. Therefore, our results suggest that the surface morphology and compound contents are obviously different between FF in August and October. In agreement with our study, Qu et al. showed that phytochemical profiles were similar among different Qingqiao and Laoqiao samples, while contents of major components are significantly different (Qu et al., 2017).

Near-infrared (NIR) spectroscopy is used to identify the authenticity of traditional Chinese Medicine (TCM), with faster speed, non-destructive properties, and no sample preparation (Balabin et al., 2011; Wang et al., 2012; Sarraguca et al., 2014; Borraz-Martinez et al., 2019; Sun et al., 2019). In our results, the cluster analysis based on the K-mean of the NIR spectrum suggested that FF was divided into two categories—FF in August and October—indicating that the main reason for the distinction of FF was the picking time. Furthermore, based on PCA and discriminant analysis, our results also showed that FF was significantly different in August and October, and FF from Henan, Shanxi, and Shaanxi in August was also different, whereas FF from different regions in October was not. Our results were in agreement with those of other studies, which reported that the green and ripe FF were clearly separated by HPLC-ESI-MS/MS and NMR-based analysis, and the FF from Henan, Shanxi, and Anhui were clearly different (Jia et al., 2015; Qu et al., 2017).

Furthermore, the levels of forsythiaside A, phillyrin, and phillygenin, the main components of FF, were analyzed by HPLC. We found that the contents of them in FF were significantly increased in August, compared with October. At the same time, the levels of forsythiaside A and phillyrin of FF from Shanxi in August and phillygenin of FF from Shaanxi in August were the highest. Previous studies have found that forsythiaside A and phillyrin exhibited anti-oxidative, anti-inflammatory, antiviral (Wei et al., 2014; Qu et al., 2016; Dong et al., 2017; Zhang et al., 2020a; Gong et al., 2020; Jiang et al., 2020; Fu et al., 2021; Zhao et al., 2021), and phillygenin showed anti-oxidative, anti-inflammatory, anti-cancer (Chang et al., 2008; He et al., 2019; Hu et al., 2020; Zhou et al., 2021b). Consequently, FF from Shanxi and Shaanxi in August was selected to study which one has better anti-oxidative and anti-inflammatory effects based on the high levels of forsythiaside A, phillyrin, and phillygenin of FF.

Excessive secretion of inflammatory cytokines, such as TNF-α, IL-1β, and inflammatory mediators, for example, NO, iNOS have been proved to lead to inflammation, and NF-κB, the key transcription factor of inflammation, regulates the secretion of inflammatory cytokines (An et al., 2020; Chen et al., 2021). In our study, our results showed that FF markedly reduced the levels of inflammatory cytokines, inflammatory mediators, and the protein expression levels of phosphorylated total IKKα/β and nuclear NF-κB, and the inhibitory effects of FF from Shaanxi on IKKα/β and NF-κB were higher than those of FF from Shanxi, indicating that the anti-inflammatory of FF from Shanxi and Shaanxi in August may be related to NF-κB signaling, and the activity of FF from Shaanxi is higher than that of FF from Shanxi. FF has been proven by many studies to alleviate diseases or LPS-induced inflammation, which is consistent with our results, but there are few studies on the comparison of anti-inflammatory effects of FF from different regions.

Keap1 negatively regulates the activation of Nrf2, and activated Nrf2 regulates the release of HO-1, NQO1, and antioxidant enzymes to play an antioxidant role, which is conducive to down-regulating inflammation (Wang et al., 2021c; Li et al., 2021; Osman et al., 2021). In the present study, the activity of Nrf2 was activated by FF, and the mRNA expression of HO-1 and NQO1 was increased, which indicated that FF may perform antioxidant action by activating the Nrf2 pathway. Consistent with the anti-inflammatory action of FF, FF from Shaanxi has a more obvious antioxidant effect than FF from Shanxi.

Forsythiaside A, phillyrin, and phillygenin have been shown to have significant anti-inflammatory and antioxidant activities in previous studies, but there are few studies on their comparison of effects (Yuan et al., 2014; Ma et al., 2019; Du et al., 2020; Zhou et al., 2021a; Tian et al., 2021). In this study, correlation analysis demonstrated that phillygenin and phillyrin, which belong to lignans, may be the main anti-inflammatory and antioxidant compounds of FF.

In conclusion, our results indicated that FF from Henan, Shanxi, and Shaanxi in August and October have different microstructures and mid-infrared and near-infrared spectrums. Our findings also shown that the levels of forsythiaside A and phillyrin of FF from Shanxi in August and phillygenin of FF from Shaanxi in August were higher than that of other regions and harvest seasons, especially FF in October, which indicated that the content of the compounds of FF in August (named Qingqiao) is higher than that of FF in October (named Laoqiao). What is more, FF from Shanxi and Shaanxi in August showed marked anti-inflammatory and antioxidant activities, but with some differences, which may be mainly due to the different content of phillygenin and phillyrin. Our finding provides a new perspective for FF research, whereas further in-depth research will be needed to prove whether phillygenin or phillyrin is the main anti-inflammatory and antioxidant component in FF.

## Data Availability

The original contributions presented in the study are included in the article/Supplementary Material, further inquiries can be directed to the corresponding authors.

## Abbreviations

FF, Forsythiae Fructus; TCM, Traditional Chinese Medicine; SEM, scanning electron microscope; MIR, Mid-infrared; NIR, Near-infrared; RT-qPCR, real-time quantitative PCR; PCA, Principal component analysis.
